# Neurotransmitter Mechanisms of Ketamine and Ketamine–Magnesium Sulfate-Induced Hypothermia: Evidence for Serotonergic and Adrenergic Involvement Without GABA_A_ Contributions

**DOI:** 10.3390/brainsci16020189

**Published:** 2026-02-04

**Authors:** Katarina Savić Vujović, Sonja Vučković, Lara Samardžić, Branislava Medić, Dragana Srebro, Ana Jotić, Ivana Ćirković

**Affiliations:** 1 Department of Pharmacology, Clinical Pharmacology and Toxicology, Faculty of Medicine, University of Belgrade, 11129 Belgrade, Serbia; vuckovicsonja1@gmail.com (S.V.); lara.samardzic27@gmail.com (L.S.); brankicamedic@gmail.com (B.M.); srebrodragana1@gmail.com (D.S.); 2 Clinic for Otorhinolaryngology and Maxillofacial Surgery, University Clinical Center of Serbia, Pasterova 2, 11000 Belgrade, Serbia; anajotic@yahoo.com; 3 Institute of Microbiology and Immunology, Faculty of Medicine, University of Belgrade, Dr Subotica 1, 11000 Belgrade, Serbia; cirkoviciv@yahoo.com

**Keywords:** ketamine, magnesium, hypothermia, rats, serotonergic system, noradrenergic system, GABAergic system

## Abstract

**Background:** Ketamine and magnesium sulfate are commonly used perioperatively to prevent shivering, a frequent and clinically relevant complication of spinal and general anesthesia. Although their hypothermic effects are well documented, the neurotransmitter mechanisms underlying these effects remain insufficiently understood. This study examines whether serotonergic, adrenergic (α_2_), and GABAergic (GABA_A_) systems contribute to hypothermia induced by ketamine and a ketamine–magnesium sulfate combination. **Methods:** Body temperature was measured in Wistar rats after administration of ketamine (10 mg/kg) or the ketamine (5 mg/kg)–magnesium sulfate (5 mg/kg) combination. To assess neurotransmitter involvement, animals received yohimbine (α_2_ antagonist), methysergide (non-selective 5-HT antagonist), or bicuculline (GABA_A_ antagonist) prior to ketamine or the drug combination. Data were analyzed using two-way repeated measures ANOVA followed by Tukey’s post hoc test. **Results:** Yohimbine at 0.5 and 1 mg/kg significantly potentiated ketamine-induced hypothermia, while only 3 mg/kg enhanced the effect of the ketamine–magnesium sulfate combination. Methysergide had a bidirectional influence: 1 mg/kg methysergide deepened ketamine-induced hypothermia, whereas 0.5 mg/kg methysergide attenuated the hypothermic effect of the ketamine–magnesium sulfate combination. Bicuculline (1–2 mg/kg) did not alter the hypothermic responses to ketamine or the combination. **Conclusions:** These findings indicate that ketamine- and ketamine–magnesium sulfate-induced hypothermia is primarily modulated by serotonergic and adrenergic mechanisms, whereas GABA_A_ receptor-dependent pathways do not appear to play a major role under the experimental conditions used. These results provide new mechanistic insights into NMDA antagonist–related thermoregulation and may help inform anesthetic strategies for shivering prevention and maintenance of perioperative thermal stability.

## 1. Introduction

Shivering is a frequent and unpleasant adverse effect following spinal anesthesia, and in high-risk patients it may contribute to additional postoperative complications [1,2,3]. Ketamine and magnesium sulfate are known to reduce this response. Both agents act as antagonists of N-methyl-D-aspartate (NMDA) receptors, and NMDA receptor blockade is associated with a reduction in body temperature and induction of hypothermia [4,5]. The glutamatergic system, acting through NMDA receptors, plays a central role in thermoregulation, and NMDA antagonists can modulate the activity of thermosensitive neurons in the hypothalamus [6].

Experimental data have demonstrated that ketamine and magnesium sulfate interact synergistically to lower body temperature, producing a clear dose-dependent hypothermic effect in rats [5]. Notably, ketamine administered at 10 mg/kg produced a hypothermic response comparable to that induced by the combination of ketamine (5 mg/kg) and magnesium sulfate (5 mg/kg). Previous research suggests that the nitric oxide (NO) pathway participates in the hypothermic action of the ketamine–magnesium sulfate combination, but not in the effect of ketamine alone. Neuronal nitric oxide synthase has been implicated in mediating the temperature-lowering effect of the combined treatment [7].

Despite these findings, the neurotransmitter mechanisms contributing to the hypothermic effects of ketamine and the ketamine–magnesium sulfate combination remain poorly characterized. Given the central role of monoaminergic and inhibitory neurotransmission in thermoregulation, the present study focuses on the adrenergic, serotonergic, and GABAergic systems as biologically plausible modulators of NMDA-antagonist–induced hypothermia. α_2_-adrenergic pathways are critically involved in the regulation of sympathetic tone and heat dissipation, whereas serotonergic signaling, particularly via 5-HT_1A_ receptors, plays a key role in hypothalamic thermoregulation. In addition, GABA_A_-mediated inhibitory mechanisms within the preoptic area and hypothalamus are essential components of central thermoregulatory circuits [4,6,8]. Therefore, these systems were selected to determine whether ketamine alone and the ketamine–magnesium sulfate combination recruit overlapping or distinct neurochemical mechanisms underlying hypothermia.

The aim of this study was to determine whether the adrenergic (α_2_), serotonergic (5-HT), and GABAergic (GABA_A_) systems participate in the hypothermic actions of ketamine (10 mg/kg) and the combination of ketamine (5 mg/kg) and magnesium sulfate (5 mg/kg).

## 2. Materials and Methods

### 2.1. Animals

All procedures were conducted in accordance with the national and international guidelines for the care and use of laboratory animals. Ethical approval was obtained from the Ethics Committee for Animal Research and Welfare of the Faculty of Medicine, University of Belgrade (No. 323-07-07783/2020-05/03) and from the Ethical Council for the Protection of Experimental Animals of the Ministry of Agriculture, Forestry and Water Management of the Republic of Serbia. This study complied with the Serbian Animal Welfare Law and the National Institutes of Health Guide for the Care and Use of Laboratory Animals (NIH Publication No. 8023, revised 1978).

A total of 168 Wistar rats (200–250 g) were used in this study. Adult male Wistar rats (8–10 weeks old, body weight 200–250 g) were used in all experiments. The animals were obtained from the Military Medical Academy breeding facility (Belgrade, Serbia). Rats were housed three per cage (42.5 × 27 × 19 cm) under controlled environmental conditions (temperature 22 ± 1 °C, relative humidity 60%, and 12 h light/dark cycle). Standard chow and water were provided ad libitum, except during the experimental sessions.

Body temperature measurements were consistently started between 08:00 and 09:00 h to minimize circadian variation. The animals were not restrained, except during the brief testing period. For each measurement, the rat was taken individually from the home cage, placed in a Plexiglas restrainer for approximately 15 s, and immediately returned afterward. This standardized handling ensured uniform and minimal distress in all animals. The experimental groups consisted of 6–8 rats, and each animal was used only once.

### 2.2. Temperature Measurement

Animals were familiarized with the handling and experimental procedures for three successive days prior to the procedure. They were acclimatized to laboratory conditions for 60 min). Temperature was measured using a calibrated rectal thermistor probe connected to an ALMEMO^®^ measuring system (Ahlborn Mess- und Regelungstechnik GmbH, Holzkirchen, Germany). The colonic temperature of the rats was taken several times. When the body temperature of the rats was stable (usually 2 h after acclimatization), we began measuring the baseline body temperature at 30 min intervals, and three control values were obtained. In the next step, drugs and/or 0.9% NaCl were administered, and the temperature was measured at 30, 60, 90, 120, 150, and 180 min after ketamine injection.

### 2.3. Drug Administration

The drugs used in this study included ketamine and magnesium sulfate (NMDA receptor antagonists), yohimbine (α_2_-adrenergic receptor antagonist), methysergide (non-selective 5-HT receptor antagonist), and bicuculline (GABA_A_ receptor antagonist). Ketamine (Inresa Arzneimittel GmbH, Freiburg, Germany), yohimbine and methysergide (Sigma-Aldrich Chemical Co., St. Louis, MO, USA), and magnesium sulfate (Zorka, Šabac, Serbia) were freshly dissolved in 0.9% NaCl. Bicuculline was initially dissolved in a minimal volume of 0.1 N HCl, diluted with 0.9% NaCl to the desired concentration, and the pH was adjusted to the physiological range (7.2–7.4) prior to administration. Ketamine was administered intraperitoneally (i.p.), whereas yohimbine, bicuculline, methysergide, and magnesium sulfate were injected subcutaneously (s.c.) in a final volume of 2 mL/kg.

Yohimbine, bicuculline, and methysergide were administered 10 min prior to ketamine, whereas magnesium sulfate was administered 5 min before ketamine. The animals in the control group received an equivalent volume of 0.9% NaCl. Pretreatment intervals were selected based on the pharmacodynamic properties and previously established experimental protocols of the administered agents to ensure adequate central receptor engagement at the time of ketamine administration.

The selected doses of yohimbine, methysergide, and bicuculline were based on previously published studies that demonstrated effective central receptor engagement without inducing marked behavioral toxicity. The dose ranges included sub-threshold to moderate concentrations commonly used to probe adrenergic, serotonergic, and GABA_A_ receptor–mediated mechanisms. Importantly, the doses were intentionally limited to minimize off-target effects while still allowing the detection of functional modulation of hypothermic responses.

### 2.4. Statistical Analysis

Data are expressed as mean ± standard error of the mean (SEM). Because body temperature was repeatedly measured in the same animals across multiple time points, the data were analyzed using a two-way repeated-measures ANOVA, with treatment as the between-subjects factor and time as the within-subjects factor, followed by Tukey’s post hoc test for multiple comparisons. This model evaluated the main effects of treatment and time, as well as the treatment × time interaction.

Statistical analysis was originally performed on raw data using GraphPad Prism (version 9.0, GraphPad Software, San Diego, CA, USA). Due to the unavailability of the original ANOVA output, F-statistics and degrees of freedom are not reported.

Before performing ANOVA, assumptions of normality and homogeneity of variances were assessed using the Shapiro–Wilk test and Levene’s test, respectively, and no violations were detected. Statistical significance was defined as *p* < 0.05.

## 3. Results

### 3.1. Effect of Yohimbine on the Hypothermic Effect of Ketamine (10 mg/kg)

Yohimbine (0.5–3 mg/kg, s.c.) did not alter basal body temperature compared with 0.9% NaCl (*p* > 0.05). However, pretreatment with yohimbine at 0.5 and 1 mg/kg significantly potentiated the hypothermic effect of ketamine (10 mg/kg, i.p.) (*p* < 0.01). The enhancement appeared within 30 min, peaked at 60 min, and persisted up to 120 min after ketamine injection. The maximal decrease in body temperature reached approximately 2.4–2.5 °C, representing an additional ~2 °C reduction relative to ketamine alone.

### 3.2. The Effect of Yohimbine on the Combination of Ketamine (5 mg/kg) and Magnesium Sulfate (5 mg/kg)

**Figure 1:**
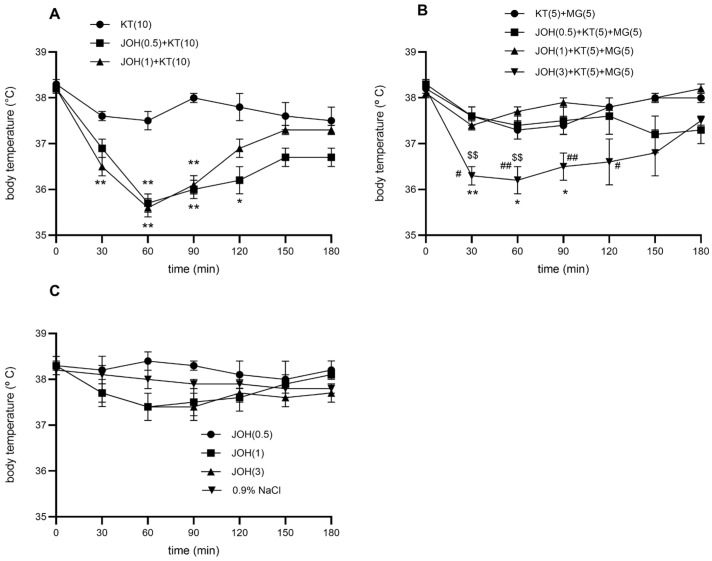
Time-response curves for: (**A**) combinations of ketamine (KT, 10 mg/kg, i.p.) and yohimbine (JOH, 0.5 and 1 mg/kg, s.c.); (**B**) combinations of ketamine (KT, 5 mg/kg, i.p.), magnesium sulfate (MG 5 mg/kg, s.c.) and yohimbine (JOH, 0.5, 1 and 3 mg/kg, s.c.); and (**C**) yohimbine (JOH, 0.5, 1 and 3 mg/kg, s.c.) to the body temperature of rats. Each point represents the mean ± SEM. There is statistical difference between: (**A**) KT (10) and the combination of JOH (0.5, 1)-KT (10) (** *p* < 0.01, * *p* < 0.05) (**B**) KT(5)-MG(5) and JOH(3)-KT(5)-MG(5) (** *p* < 0.01, * *p* < 0.05); JOH(1)-KT(5)-MG(5) and JOH(3)-KT(5)-MG(5) (## *p* < 0.01, # *p* < 0.05); JOH(0.5)-KT(5)-MG(5) and JOH(3)-KT(5)-MG(5) ($$ *p* < 0.01).

At doses of 0.5 and 1 mg/kg, yohimbine did not alter the hypothermic effect produced by the ketamine–magnesium sulfate combination. In contrast, 3 mg/kg yohimbine significantly enhanced the hypothermia induced by the combination. The ketamine–magnesium sulfate treatment alone decreased body temperature by approximately 0.7 °C, whereas co-administration with yohimbine at 0.5, 1, and 3 mg/kg resulted in reductions of 0.6 °C, 0.7 °C, and 1.8 °C, respectively, relative to the saline group. At the highest dose (3 mg/kg), yohimbine further deepened the hypothermic effect by an additional 0.9 °C compared with the ketamine–magnesium sulfate combination alone. A statistically significant difference was observed between the yohimbine 1 mg/kg + ketamine–magnesium sulfate group and the yohimbine 3 mg/kg + ketamine–magnesium sulfate group. (## *p* < 0.01, # *p* < 0.05), as well as between the combinations of yohimbine(0.5)-ketamine(5)-magnesium sulfate(5) and yohimbine (3)-ketamine (5)-magnesium sulfate (5) ($$ *p* < 0.01, $ *p* < 0.05) (Figure 1C).

### 3.3. The Effect of Methysergide on the Hypothermic Effect of Ketamine (10 mg/kg)

**Figure 2:**
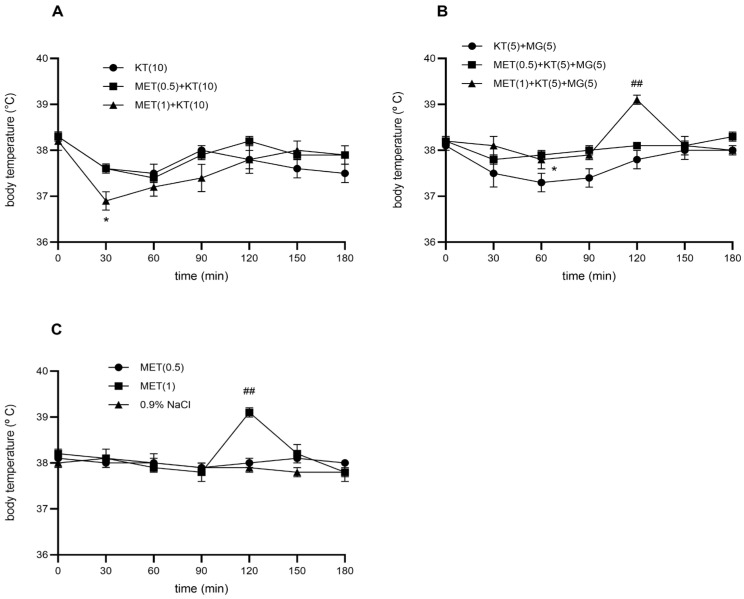
Time-response curves for: (**A**) combinations of ketamine (KT, 10 mg/kg, i.p.) and methysergide (MET, 0.5 and 1 mg/kg, s.c.); (**B**) combinations of ketamine (KT, 5 mg/kg, i.p.), magnesium sulfate (MG, 5 mg/kg, s.c.) and methysergide (MET, 0.5 and 1 mg/kg, s.c.); and (**C**) methysergide (MET, 0.5 and 1 mg/kg, s.c.) to the body temperature of rats. Each point represents the mean ± SEM. There is statistical difference between: (**A**) KT (10) and the combination of MET(1)-KT (10) (* *p* < 0.05) (**B**) KT(5)-MG(5) and MET(0.5)-KT(5)-MG(5) (* *p* < 0.05); KT(5)-MG(5) and MET(1)-KT(5)-MG(5) (## *p* < 0.01) (C) between MET (1) and 0.9% NaCl (## *p* < 0.01).

Methysergide at 0.5 and 1 mg/kg did not alter basal body temperature compared with 0.9% NaCl (*p* > 0.05), except that the 1 mg/kg dose produced mild hyperthermia at 120 min (Figure 2C). When combined with ketamine (10 mg/kg), the 0.5 mg/kg dose of methysergide did not modify the hypothermic response (*p* > 0.05). Dose of 1 mg/kg significantly potentiated ketamine-induced hypothermia (*p* < 0.05) (Figure 2A). This enhancement appeared within 30 min of ketamine administration and increased the overall hypothermic effect. Compared to saline, methysergide at 0.5 and 1 mg/kg in combination with ketamine produced maximal temperature reductions of 0.6 °C and 1.2 °C, respectively. Compared with ketamine alone, methysergide (1 mg/kg) further decreased body temperature by an additional 0.7 °C.

### 3.4. The Effect of Methysergide on the Combination of Ketamine (5 mg/kg) and Magnesium Sulfate (5 mg/kg)

At a dose of 0.5 mg/kg, methysergide antagonized the effect of the combination of ketamine (5 mg/kg) and magnesium sulfate (5 mg/kg) on body temperature at 60 min (*p* < 0.05) when it achieved the maximum effect. At a dose of 1 mg/kg, methysergide reduced the hypothermia caused by the ketamine–magnesium sulfate combination, but without statistical significance. An exception was at 120 min, when the methysergide-ketamine-magnesium sulfate combination led to hyperthermia (Figure 2C). Compared to 0.9% NaCl, at doses of 0.5 and 1 mg/kg, methysergide in combination with ketamine-magnesium sulfate reduced the body temperature by 0.6 °C (Figure 2C). In comparison to the ketamine-magnesium sulfate combination, doses of 0.5 and 1 mg/kg methysergide reduced the hypothermic effect of this combination by a maximum of 0.6 °C.

### 3.5. The Effect of Bicuculline on the Hypothermic Effect of Ketamine (10 mg/kg)

**Figure 3:**
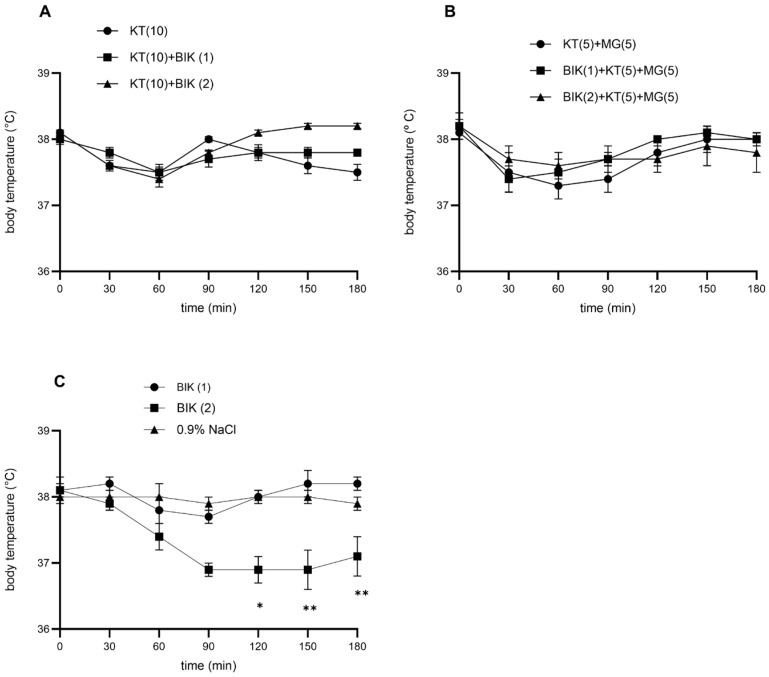
Time-response curves for: (**A**) combinations of ketamine (KT, 10 mg/kg, i.p.) and bicuculline (BIK, 1 and 2 mg/kg, s.c.); (**B**) combinations of ketamine (KT, 5 mg/kg, i.p.), magnesium sulfate (MG, 5 mg/kg, s.c.) and bicuculline (BIK, 1 and 2 mg/kg, s.c.); (**C**) bicuculline (BIK, 1 and 2 mg/kg, s.c.) on body temperature of rats. Each point represents the mean ± SEM. There is statistical difference between (**C**) BIK (2) and 0.9% NaCl (** *p* < 0.01, * *p* < 0.05).

Bicuculline (1 mg/kg) did not affect the basal body temperature compared with 0.9% NaCl (*p* > 0.05). In contrast, 2 mg/kg bicuculline induced significant hypothermia, first evident at 90 min and persisting for 180 min (*p* < 0.05) (Figure 3C). The maximal decrease in basal temperature at this dose was 1.1 °C relative to saline (*p* < 0.05).

When combined with ketamine (10 mg/kg, i.p.), bicuculline at both 1 and 2 mg/kg failed to modify the hypothermic response, with no statistically significant differences compared to ketamine alone (*p* > 0.05) (Figure 3A).

### 3.6. The Effect of Bicuculline on the Combination of Ketamine (5 mg/kg) and Magnesium Sulfate (5 mg/kg)

Bicuculline (1 or 2 mg/kg), when administered together with ketamine (5 mg/kg) and magnesium sulfate (5 mg/kg), did not modify the hypothermic effect of the ketamine–magnesium sulfate combination. No statistically significant differences were observed when compared with the combination alone (*p* > 0.05). (Figure 3B). Bicuculline at 1 mg/kg did not affect basal body temperature compared with 0.9% NaCl (*p* > 0.05). In contrast, 2 mg/kg bicuculline induced significant hypothermia, first evident at 90 min and persisting through 180 min (*p* < 0.05) (Figure 3C). The maximal decrease in basal temperature at this dose was 1.1 °C relative to saline (*p* < 0.05).

## 4. Discussion

### 4.1. Adrenergic Modulation: Effects of Yohimbine

In our previous work using a comparable experimental design, ketamine administered at 10 mg/kg consistently induced hypothermia in rats, and a low-dose combination of ketamine (5 mg/kg) and magnesium sulfate (5 mg/kg) likewise produced a reliable hypothermic response [5,7]. The present protocol employed similarly controlled ambient conditions, standardized handling, and repeated rectal temperature measurements, enabling reliable detection of modest but consistent hypothermic responses at this dose. Consistent methodology likely results in agreement between the present findings and our earlier report, while there are discrepancies in results from other studies using different experimental conditions.

Yohimbine at low doses (0.5–3 mg/kg) did not alter baseline body temperature, which aligns with previous findings [9]. However, when administered prior to ketamine (10 mg/kg), yohimbine at 0.5 and 1 mg/kg significantly potentiated ketamine-induced hypothermia, whereas these same doses did not affect the hypothermic action of the ketamine–magnesium sulfate combination. Only the highest tested dose (3 mg/kg) further deepened the hypothermia produced by the drug combination. Importantly, at this dose, yohimbine should not be regarded as a selective α_2_-adrenergic antagonist, as it is known that serotonergic activity and broader effects on monoamine release are likely to contribute to the observed hypothermic response.

Together, these findings are consistent with role of α_2_-adrenergic mechanisms in facilitating heat loss in the context of NMDA receptor inhibition, while also highlighting the significant contribution of serotonergic pathways at higher yohimbine doses. Higher doses of yohimbine are known to decrease body temperature via enhanced serotonergic outflow [10], and the drug’s affinity for 5-HT_1A_ receptors provides an additional mechanism by which it can promote hypothermia [10,11,12]. The attenuation of hypothermic responses by the selective 5-HT_1A_ antagonist WAY-100135 supports the involvement of serotonergic signaling in thermoregulation [13,14].

One possible hypothesis to explain the differential effects of yohimbine on ketamine versus ketamine–magnesium sulfate-induced hypothermia is that magnesium sulfate may modulate monoaminergic neurotransmission, including norepinephrine and serotonin release. This interpretation is based on indirect pharmacological interaction patterns and prior literature suggesting that magnesium can influence presynaptic neurotransmitter release [15]. In the absence of direct neurochemical measurements, this proposed mechanism remains speculative and should be regarded as a hypothesis rather than a definitive conclusion. Future studies incorporating neurochemical approaches, such as microdialysis, will be necessary to directly verify this assumption. Overall, the potentiation of hypothermia by yohimbine in the present study is best interpreted as reflecting combined adrenergic–serotonergic modulation rather than selective α_2_-adrenergic antagonism.

### 4.2. Serotonergic Regulation: Effects of Methysergide

Methysergide is a pharmacologically complex, non-selective serotonergic antagonist, whose effects on thermoregulation depend on the dose, receptor subtype involvement, and experimental context [16,17,18]. In addition to antagonizing 5-HT_2_ receptors, methysergide interacts functionally with serotonergic pathways implicated in heat loss and metabolic suppression, including those associated with 5-HT_1A_ signaling [19]. Moreover, methysergide-induced hyperthermia observed at higher doses or at delayed time points has been attributed to prostaglandin-dependent mechanisms [20]. These diverse pharmacodynamic effects provide a plausible explanation for the bidirectional, time-dependent findings in the present study and may clarify why ketamine-induced hypothermia differs with ketamine alone versus the ketamine–magnesium sulfate combination [21].

In the present experiments, methysergide displayed a clear dose- and context-dependent influence on thermoregulation. While the 0.5 mg/kg dose did not alter baseline body temperature, the 1 mg/kg dose produced late-onset hyperthermia, consistent with previous reports attributing methysergide-induced warming to prostaglandin release. In combination with ketamine (10 mg/kg), methysergide behaved bidirectionally, with the lower dose having no effect and the higher dose significantly enhancing hypothermia. In contrast, when combined with ketamine–magnesium sulfate, methysergide at 0.5 mg/kg antagonized the hypothermic response, whereas the 1 mg/kg dose attenuated the effect without reaching statistical significance and produced hyperthermia at later time points.

These findings suggest that serotonergic mechanisms contribute to the hypothermic effects of the ketamine–magnesium sulfate combination, potentially involving 5-HT_1A_-related pathways. However, the involvement of specific serotonergic receptor subtypes, including 5-HT_1A_ receptors, was based on pharmacological interaction patterns and prior literature and cannot be directly demonstrated within the scope of the present study. Given the non-selective pharmacological profile of methysergide, the observed effects were most appropriately interpreted as reflecting the modulation of multiple serotonergic receptor subtypes rather than the action of a single receptor population.

### 4.3. GABAergic System: Lack of Involvement of GABA

Although bicuculline at 2 mg/kg produced mild hypothermia when administered alone, it did not influence the hypothermic effects of either ketamine or the ketamine–magnesium sulfate combination. Previous studies have shown that GABAergic modulation can affect body temperature under specific experimental conditions and that functional interactions between GABAergic and serotonergic systems exist within hypothalamic thermoregulatory nuclei [22,23,24]. However, in the present model and within the tested dose range, the hypothermia observed following bicuculline administration most likely reflects a direct disruption of inhibitory tone within central thermoregulatory circuits rather than participation in NMDA antagonist–mediated mechanisms. The absence of any interaction between bicuculline and ketamine or ketamine–magnesium sulfate suggests that GABA_A_-dependent pathways do not play a major role in mediating NMDA antagonist–induced hypothermia under the experimental conditions used in this study.

### 4.4. Clinical Relevance and Implications for Perioperative Medicine

Human perioperative studies have provided direct clinical evidence supporting the anti-shivering efficacy of both ketamine and magnesium sulfate. In a randomized, double-blind clinical trial, prophylactic low-dose ketamine infusion significantly reduced the incidence and severity of shivering during spinal anesthesia compared to placebo [25]. A recent randomized clinical study demonstrated that intravenous magnesium sulfate dose-dependently decreased the incidence of post-subarachnoid block shivering, confirming its clinical utility in neuraxial anesthesia settings [26]. Ketamine and magnesium sulfate are widely used in anesthesia because of their analgesic, sympatholytic, and anti-shivering properties. Understanding how monoaminergic pathways modulate their thermoregulatory effects is critical, as excessive hypothermia may impair postoperative recovery, increase oxygen consumption, and compromise cardiovascular stability.

Our results indicate that agents acting on α_2_ or 5-HT receptors may significantly alter the temperature-lowering effects of ketamine and ketamine–magnesium sulfate. This highlights the importance of carefully selecting adjunct medications during anesthesia and sedation to avoid the unintended deepening of hypothermia or disruption of thermal homeostasis.

This study compared ketamine alone and the ketamine–magnesium sulfate combination within the same experiment. The dose-dependent modulation observed with yohimbine and methysergide indicates that these two hypothermic paradigms, although both involving NMDA receptor antagonism, partially recruit different monoaminergic mechanisms.

This study has several limitations. First, no direct neurochemical measurements (e.g., microdialysis) were performed to confirm the changes in monoaminergic neurotransmission. Second, this study relied on pharmacological antagonists, which may exert off-target effects despite careful dose selection. Finally, only male rats were used, and potential sex-dependent differences in thermoregulatory responses were not addressed. These limitations should be considered when interpreting the findings and highlight the directions for future research.

## 5. Conclusions

The present study demonstrates that hypothermia induced by ketamine and by the ketamine–magnesium sulfate combination is predominantly modulated by monoaminergic mechanisms, with serotonergic and adrenergic pathways playing a central role, whereas GABA_A_ receptor–dependent mechanisms do not appear to substantially contribute under the experimental conditions employed. The differential modulation observed with yohimbine and methysergide indicates that, although both hypothermic paradigms involve NMDA receptor antagonism, ketamine alone and the ketamine–magnesium sulfate combination partially recruit distinct monoaminergic regulatory mechanisms.

The hypothermic response to the ketamine–magnesium sulfate combination was particularly sensitive to serotonergic modulation. Pharmacological interaction patterns observed with methysergide are consistent with the involvement of multiple serotonergic receptor subtypes and are compatible with a contribution of 5-HT_1A_ related signaling; however, the participation of specific receptor populations cannot be directly established within the scope of the present study. Similarly, potentiation of hypothermia by yohimbine supports an important modulatory role of α_2_ adrenergic and serotonergic mechanisms, especially at higher doses where yohimbine exerts non-selective monoaminergic effects.

Taken together, these findings advance the understanding of neurochemical regulation of thermogenesis during NMDA receptor inhibition and emphasize the importance of monoaminergic modulation in shaping ketamine- and magnesium-related alterations in body temperature. By delineating the neurotransmitter systems that influence these effects, the present study provides experimental insight that may help inform the rational selection of adjunct anesthetic and analgesic agents aimed at minimizing shivering while preserving perioperative thermal homeostasis.

## Data Availability

The original contributions presented in this study are included in the article. Further inquiries can be directed to the corresponding author.

## Abbreviations

The following abbreviations are used in this manuscript:

NMDA	N-methyl-D-aspartate
GABA	gamma-aminobutyric acid
YOH	yohimbine
MET	Methysergide
BIK	bicuculline
KT	ketamine
MG	magnesium
